# Effects of Different Tillage and Straw Return on Soil Organic Carbon in a Rice-Wheat Rotation System

**DOI:** 10.1371/journal.pone.0088900

**Published:** 2014-02-20

**Authors:** Liqun Zhu, Naijuan Hu, Minfang Yang, Xinhua Zhan, Zhengwen Zhang

**Affiliations:** 1 College of Agriculture, Nanjing Agricultural University, Nanjing, China; 2 College of Resources and Environmental Science, Nanjing Agricultural University, Nanjing, China; North Carolina State University, United States of America

## Abstract

Soil management practices, such as tillage method or straw return, could alter soil organic carbon (C) contents. However, the effects of tillage method or straw return on soil organic C (SOC) have showed inconsistent results in different soil/climate/cropping systems. The Yangtze River Delta of China is the main production region of rice and wheat, and rice-wheat rotation is the most important cropping system in this region. However, few studies in this region have been conducted to assess the effects of different tillage methods combined with straw return on soil labile C fractions in the rice-wheat rotation system. In this study, a field experiment was used to evaluate the effects of different tillage methods, straw return and their interaction on soil total organic C (TOC) and labile organic C fractions at three soil depths (0–7, 7–14 and 14–21 cm) for a rice-wheat rotation in Yangzhong of the Yangtze River Delta of China. Soil TOC, easily oxidizable C (EOC), dissolved organic C (DOC) and microbial biomass C (MBC) contents were measured in this study. Soil TOC and labile organic C fractions contents were significantly affected by straw returns, and were higher under straw return treatments than non-straw return at three depths. At 0–7 cm depth, soil MBC was significantly higher under plowing tillage than rotary tillage, but EOC was just opposite. Rotary tillage had significantly higher soil TOC than plowing tillage at 7–14 cm depth. However, at 14–21 cm depth, TOC, DOC and MBC were significantly higher under plowing tillage than rotary tillage except for EOC. Consequently, under short-term condition, rice and wheat straw both return in rice-wheat rotation system could increase SOC content and improve soil quality in the Yangtze River Delta.

## Introduction

Soil organic carbon (C) has profound effects on soil physical, chemical and biological properties [1]. Maintenance of soil organic C (SOC) in cropland is important, not only for improvement of agricultural productivity but also for reduction in C emission [2]. However, short- and medium-term changes of SOC are difficult to detect because of its high temporal and spatial variability [3]. On the contrary, soil labile organic C fractions (i.e., microbial biomass C (MBC), dissolved organic C (DOC), and easily oxidizable C (EOC)) that turn over quickly can respond to soil disturbance more rapidly than total organic C (TOC) [1], [3], [4]. Therefore, these fractions have been suggested as early sensitive indicators of the effects of land use change on soil quality (e.g. [3], [5], [6]).

Agricultural practices such as tillage methods are conventionally used for loosening soils to grow crops. But long-term soil disturbance by tillage is believed to be one of the major factors reducing SOC in agriculture [7]. Frequent tillage may destroy soil organic matter (SOM) [8] and speed up the movement of SOM to deep soil layers [9]. As a consequence, agricultural practices that reduce soil degradation are essential to improve soil quality and agricultural sustainability. Crop residue plays an important role in SOC sequestration, increasing crop yield, improving soil organic matter, and reducing the greenhouse gas (e.g. [10]–[13]). As an important agricultural practice, straw return is often implemented with tillage in the production process. Although numerous studies have indicated that tillage methods combined with straw return had a significant effect on labile SOC fractions, the results varied under different soil/climate conditions. For example, both no-tillage and shallow tillage with residue cover had significantly higher SOC than conventional tillage without residue cover in Loess Plateau of China [14], while Wang et al. [15] reported that the difference between the treatments of plowing with straw return and no-tillage with straw return on TOC in central China was not significant. Rajan et al. [2] showed that in Chitwan Valley of Nepal, no-tillage with crop residue application at upper soil depth had distinctly higher SOC sequestration than conventional tillage with crop residue. The effects of tillage on soil labile organic C vary with regional climate [16], soil condition (e.g. [17]–[20]), residue management practice, and crop rotation (e.g. [21], [22]). Therefore, the investigation on soil labile organic C for specific soil, climate, and cropping system is necessary to improve the soil quality.

The Yangtze River Delta of China is the main production region of rice and wheat, and rice-wheat rotation is the most important cropping system in this region [23]. The total sown area of rice and wheat in the Yangtze River Delta accounted for about 20.1% of that in China in 2011, and the total yield was 22.1% of the national yield for these two crops [24]. Many field experiments in this region (e.g. [25]–[27]) about the effects of tillage methods combined with straw return on cropland ecosystem in rice-wheat rotation system have been studied during these years. However, most of them are focused on soil physical-chemical properties, soil nutrient and crop yield. To our knowledge, there is little information about the effects of different tillage and straw return on soil labile C fractions in rice-wheat rotation system. Thus, the objectives of this study were (1) to quantify the effects of tillage methods and straw return on soil TOC, MBC, DOC, EOC contents in the rice-wheat rotation system in the Yangtze River Delta, and (2) to explore an optimal management practice combination of tillage and straw return for improving the soil quality and increasing the local crop production.

## Materials and Methods

### Site Description

The experiment was conducted at Changwang Country, Youfang Town, Yangzhong City, Jiangsu Province, China (119°42′–119°58′E, 32°–2°19′N, 4–4.5 m above mean sea level) from November, 2009 to June, 2011. Access to the study site was obtained in the form of a rent contract, in which we had to confirm that our study did not involve endangered or protected species.

The experimental site had a subtropical monsoon climate with an average annual precipitation of 1000 mm, an average annual temperature of 15.1°C, and a mean annual sunshine hour of 2135 h. The soil of the experimental site was a loam and classified as an anthrosols. Rice-wheat double cropping system was the most important cropping system in the region. The main properties of soil (0–20 cm depth) sampled in November 2009 were as follows: soil organic matter 29.81 g kg^−1^; alkali-hydrolyzale nitrogen 194.02 mg kg^−1^; available phosphorus 13.60 mg kg^−1^; available potassium 51.45 mg kg^−1^; and pH 7.34.

The variety of wheat used in this study was Yangmai16 *(Triticum aestivum* L.*)* and rice was Nangeng47 (*Oryza sativa* L.).

### Experimental Design and Field Managements

The experiment had a split-plot design with two tillage methods in the main plots and four straw return modes in subplots with three replications (6 m×5 m). Tillage methods included plowing tillage (P) and rotary tillage (R). Straw return modes were as follows: no straw return (N), only rice straw return (R), only wheat straw return (W), and rice and wheat straw both return (D). There were eight treatments in this study: (1) plowing tillage with no straw return (PN: rice with plowing tillage-wheat with plowing tillage); (2) plowing tillage with only rice straw return (PR: rice with plowing tillage - wheat with plowing tillage+rice straw return); (3) plowing tillage with only wheat straw return (PW: rice with plowing tillage+wheat straw return - wheat with plowing tillage); (4) plowing tillage with rice and wheat straw both return(PD: rice with plowing tillage+wheat straw return -wheat with plowing tillage+rice straw return ); (5) rotary tillage with no straw return (RN: rice with rotary tillage - wheat with rotary tillage); (6) rotary tillage with only rice straw return (RR: rice with rotary tillage - wheat with rotary tillage+rice straw return); (7) rotary tillage with only wheat straw return (RW: rice with rotary tillage+wheat straw return - wheat with rotary tillage); (8) rotary tillage with rice and wheat straw both return (RD: rice with rotary tillage+wheat straw return - wheat with rotary tillage+rice straw return).

The experimental site was cultivated with a rice-wheat rotation prior to November 2009, where wheat was planted with plowing tillage from November to the following June, and rice was transplanted by plowing tillage from June to November. In this study, after wheat or rice was harvested, they were cultivated at a depth of 10–15 cm by rotary cultivation in rotary tillage plots while for the plowing tillage plots, cultivation was at a depth of 20–25 cm with a moldboard plough. Before the rice and wheat were sown, the plowing tillage plots were disked and moldboard plowed for weed control and bedding. This was followed by an application of fertilizer. For straw returned plots, the wheat and rice straw were cut into 8–10 cm after air-dried, and placed back on the surface of the soil in June or November of each year, with returned amount of 6000 kg·hm^−2^ for both wheat and rice straw.

In this study, wheat was sown on November 3, 2009 and November 24, 2010, respectively. The seed quantity was 150 kg hm^−1^ by machine. The base fertilizer applied before sowing was 135 kg·hm^−2^ pure N, 67.5 kg·hm^−2^ P_2_O_5_, and 67.5 kg·hm^−2^ K_2_O, and topdressing was at the elongation stage with 135 kg hm^−1^ pure N. For all treatments, N was applied in the form of CO(NH_2_)_2_, and the fertilizer in wheat seasons was applied at the same rate. The wheat was harvested on June 3, 2010 and June 8, 2011, respectively. Rice was transplanted at about 3–4 seedlings per hole, 255,000 holes per hectare on June 15, 2010 and June 15, 2011, respectively. The rice was fertilized just before transplanting with a base fertilizer (120 kg hm^−1^ pure N; 60 kg hm^−1^ P_2_O_5_; 60 kg hm^−1^ K_2_O), and at tillering stage and earing stage with a topdressing (180 kg hm^−1^ N (3∶1 ratio)). The fertilizer applied in the two rice seasons were the same. The rice was harvested on November 12, 2010 and November 29, 2011, respectively. The pesticide management of both rice and wheat seasons was in accordance with the conventional, and all other management procedures were identical for the eight treatments.

### Soil Sampling and Analytical Methods

Soil samples were collected by a geotome (5 cm diameter) on October 29, 2011 (just before the rice was harvested). Five random locations were chosen in each of the 24 observational plots and samples were taken from each location at three soil depths (0–7, 7–14, and 14–21 cm) separately. Soil samples from each depth were about 200 g, fully blended. The collected moist samples were ground and sieved through a 10 mesh screen. Sieved soil samples were divided into two sub-samples. One was air-dried and sieved again through 100 mesh screen for determining soil TOC and EOC. Another was immediately stored in 4°C refrigerators for determining DOC and MBC. During sieving, crop residues, root material and stones were removed.

Total organic C (TOC) concentration was determined by oxidation with potassium dichromate and titration with ferrous ammonium sulphate [28].

Dissolved organic C (DOC) was extracted from 10 g of moist soil with 1∶2.5 ratio of soil to water at 25.8°C [29]. After shaking for 1 h and centrifuging for 10 min at 4500 r min^−1^, the supernatant was filtered with a 0.45 mm membrane filter. The filtrate was measured by oxidation with potassium dichromate and titration with ferrous ammonium sulphate.

Microbial biomass C (MBC) was analyzed by the fumigation extraction method [30]. Each sample was weighed into two equivalent portions, one was fumigated for 24 h with ethanol-free chloroform and the other was the unfumigated control. Both fumigated and unfumigated soils were shaken for 1 h with 0.5 M K_2_SO_4_ (2∶5 soil: extraction ratio), centrifuged and filtered.

Easily oxidizable C (EOC) was measured as described by Blair et al. [3]. Finely ground air-dried soil samples were reacted with 333 mmol L^−1^ KMnO_4_ by shaking at 60 r min^−1^ for 1 h. The suspension was then centrifuged at 2000 r min^−1^ for 5 min. The supernatant was diluted and measured spectrophotometrically at 565 nm. All soil samples were analyzed in triplicate.

### Data Analysis

The SPSS 16.0 analytical software package was used for all statistical analyses. A 2-factor analysis of variance (ANOVA) was employed for difference test among eight treatments at *P*<0.05, with separation of means by least significant difference (LSD). Correlation analysis were performed to determine correlations among soil labile organic C fractions in the 0–21 cm soil depth, and the significant probability levels of the results were given at the *P*<0.05 (*) and *P*<0.01 (**), respectively. Moreover, the affecting force analysis of tillage factor, straw factor and their interaction influence on labile organic C fractions was calculated based on the method of Leng [31]: the affecting force of tillage = tillage variables (square)/total variables (sum of total squares)×100%; the affecting force of straw return = straw return variables (square)/total variable (sum of total squares)×100%; the affecting force of interaction = interaction variable (square)/total variables ((total squares of sum)×100%.

## Results

### Soil TOC, DOC, MBC and EOC Contents in Different Treatments

**Figure 1:**
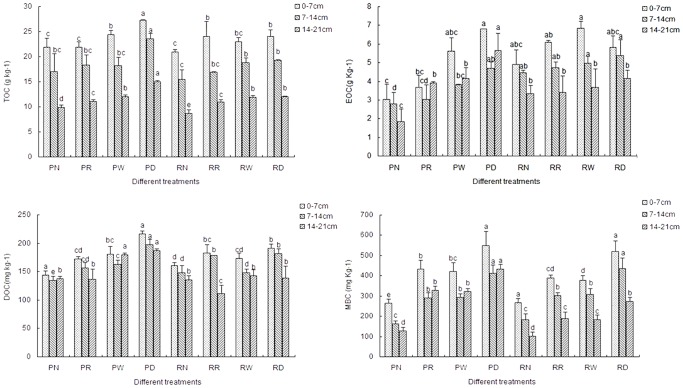
Effects of eight treatments on soil TOC, EOC, DOC and MBC contents at three depths.

As shown in Fig. 1, the different treatments significantly affected the contents of soil TOC and labile organic C fractions, where PD generally had the highest contents of TOC, DOC, MBC and EOC at the three soil depths. Crop straw return treatments (PR, PW, PD, RR, RW, RD) had consistently higher amount of TOC and labile organic C fractions at the three soil depths than without crop straw return treatments (PN, RN). Moreover, PN had significantly lower TOC, DOC, MBC and EOC at 0–7 cm and 7–14 cm, and RN had the lowest TOC and MBC at 14–21 cm compared to other treatments (Fig. 1). Soil TOC and labile organic C fractions generally decreased with an increase in soil depth under all treatments. As expected, soil TOC and labile organic C fractions were significantly and positively correlated with each other (Table 1).

**Table 1:** Linear correlations among soil TOC and labile organic C fractions at the 0–21 cm depth.

Index	TOC	EOC	DOC	MBC
TOC	1			
EOC	0.638**	1		
DOC	0.758**	0.684**	1	
MBC	0.741**	0.639**	0.908**	1

TOC: total organic carbon; MBC: microbial biomass carbon; DOC: dissolved organic carbon; EOC: easily oxidizable carbon.

* *P*<0.05.

**P<0.01.

### Effects of Different Tillage Methods on Soil TOC and Labile Organic C Fractions

Tillage had a significant effect on MBC at 0–7 cm soil depth, but seldom on soil TOC, DOC and EOC. Soil TOC, DOC and MBC contents were all higher under plowing tillage (P) than rotary tillage (R), while EOC was opposite at 0–7 cm soil depth (Table 2). At 7–14 cm, soil EOC under rotary tillage (R) was significantly higher than plowing tillage (P), but TOC had the contrary results, and there were no significant differences on DOC and MBC (Table 2). At 14–21 cm, soil TOC, DOC and MBC were significantly higher under plowing tillage (P) than rotary tillage (R), except EOC (Table 2).

**Table 2:** Effects of different tillage factor and straw factor on soil TOC, EOC, DOC and MBC at 0–7 cm, 7–14 cm and 14–21 cm depth.

Soil dept(cm)	Factors	Treatments	TOC(g kg^−1^)	labile organic carbon fractions contents		
				EOC (g kg^−1^)	DOC(mg kg^−1^)	MBC(mg kg^−1^)
0–7	Tillage factor	P	23.87±2.67a	4.78±1.23a	178.36±33.74a	417.16±17.20a
		R	23.00±2.38a	5.92±1.48a	177.05±30.96a	389.22±36.56b
	Straw factor	N	21.40±1.87c	3.97±0.92b	152.25±10.76c	266.73±11.68c
		R	23.01±2.97bc	4.88±1.40ab	177.61±33.57b	411.67±27.32b
		W	23.65±1.52b	6.23±1.56a	176.92±30.87b	398.57±27.34b
		D	25.68±2.63a	6.32±1.68a	204.04±22.67a	535.79±20.31a
7–14	Tillage factor	P	19.30±1.23a	3.58±1.76b	163.05±37.30a	311.42±43.89a
		R	17.64±2.48b	4.89±1.52a	164.11±27.23a	306.50±36.09a
	Straw factor	N	16.28±3.20c	3.62±1.76c	141.49±18.49d	172.63±16.58c
		R	17.63±2.86bc	3.90±2.70bc	167.39±21.63b	315.19±20.00b
		W	18.50±1.75b	4.39±0.54ab	155.47±22.41c	313.93±14.31b
		D	21.47±2.02a	5.04±0.76a	189.98±17.71a	434.09±11.65a
14–21	Tillage factor	P	12.00±2.56a	3.89±1.42a	160.06±30.21a	280.68±58.67a
		R	10.90±2.61b	3.64±1.14a	132.00±25.31b	187.08±44.86b
	Straw factor	N	9.28±1.69d	2.58±1.18c	136.34±11.66b	114.99±18.05d
		R	10.99±1.96c	3.66±1.26b	123.67±29.32c	239.95±39.07b
		W	11.97±3.06b	3.91±1.39b	161.19±25.91a	238.92±35.06c
		D	13.56±1.75a	4.90±0.68a	162.93±26.49a	341.67±31.14a

Different letters in a line under a specific influence factor denote significant difference at the 5% level. Different capitals in a column at different soil depths and treatments present significant different at the 0.05 level. TOC: total organic carbon; MBC: microbial biomass carbon; DOC: dissolved organic carbon; EOC: easily oxidizable carbon.

### Effects of Different Straw Return on Soil TOC and Labile Organic C Fractions

Straw return had significant effects on soil TOC and labile organic C at the three depths as shown in Table 2. In general, soil TOC and three labile organic C ranged in the following order: rice and wheat straw both return>only wheat or rice straw return>no straw return at three depths (Table 2). At 7–14 cm depth, only rice straw return in the wheat season had significantly higher DOC than only wheat straw return in the rice season (Table 2). However, at 14–21 cm depth, except for MBC, soil TOC, EOC, DOC under only rice straw return in the wheat season were lower than only wheat straw return in the rice season (Table 2). Moreover, there were significant differences in TOC and MBC among the four straw return at 14–21 cm depth (Table 2).

### Affecting Force Analysis of Different Tillage, Straw Return and their Interaction on Soil TOC and Labile Organic C Fractions

Affecting force of different tillage, straw return and their interaction on soil TOC and labile organic C were different with increasing soil depth (Table 3). The affecting force of tillage increased with the increase of soil depth (Table 3). Tillage had significant affecting force on EOC and MBC at 0–7 cm depth, but seldom on TOC and DOC (Table 3). At 7–14 cm depth, the affecting force of tillage on EOC was lower than at 0–7 cm, and there was no significant affecting force on soil TOC and other labile organic C fractions (Table 3). At 14–21 cm depth, tillage had significant affecting force on DOC and MBC. However, there was no significant affecting force on TOC and EOC (Table 3).

**Table 3:** Affecting force analysis of different tillage and straw return and their interaction on soil TOC, EOC, DOC and MBC at 0–7 cm, 7–14 cm and 14–21 cm depth.

Soil depth(cm)	Difference source	Affecting force(%)			
		TOC	EOC	DOC	MBC
0–7	Block	10.35	8.53	6.55	0.26
	Tillage	0.34	28.06**	0.64	2.06**
	Straw return	19.69*	24.79**	38.58**	95.95**
	Straw return × tillage	10.71	4.34	4.86	0.93
	Error	58.92	34.28	49.37	0.79
7–14	Block	19.97**	5.36	11.53	0.29
	Tillage	0.02	13.03*	0.85	0.07
	Straw return	21.41**	13.16	39.21**	97.83**
	Straw return × tillage	26.30**	3.89	11.12*	0.96
	Error	32.32	64.56	37.28	0.86
14–21	Block	10.22	1.61	16.66**	0.27
	Tillage	1.05	3.21	11.77**	23.81**
	Straw return	32.35**	25.06**	35.65**	70.17**
	Straw return × tillage	14.72*	13.95*	9.60*	4.93**
	Error	41.66	56.17	26.33	0.82

The affecting force of tillage = tillage variables (square)/total variables (sum of total squares) ×100%; the affecting force of straw = straw variables (square)/total variable (sum of total squares) ×100%; the affecting force of the interaction = interaction variable (square)/total variables ((total squares of sum) ×100%. TOC: total organic carbon; MBC: microbial biomass carbon; DOC: dissolved organic carbon; EOC: easily oxidizable carbon.

* *P*<0.05.

**P<0.01.

Straw return had significant affecting force on soil TOC, DOC and MBC at the three depths, but there was no significant affecting force on EOC at 7–14 cm (Table 3). Among the four indictors, the affecting force of straw return on MBC was the greatest at the three depths, which reached 95.95%, 97.83% and 70.17%, respectively (Table 3).

The affecting force of the interaction generally increased with an increase in soil depth (Table 3). At 0–7 cm soil depth, the interaction had no significant affecting force on soil TOC and labile organic C (Table 3). Soil TOC and DOC were mainly dominated by the interaction at 7–14 cm depth, but there was no significant affecting force on MBC and EOC. At 14–21 cm depth, the interaction had significant affecting force on soil TOC and all labile organic C (Table 3).

## Discussion

Suitable soil tillage practice can increase the SOC content, and improve SOC density of the plough layer [32]. The effect size of tillage methods on SOC dynamics depends on the tillage intensity [33]. Compared to conventional tillage (CT), no-tillage and reduced tillage could significantly improve the SOC content in cropland. Frequent tillage under CT easily exacerbate C-rich macroaggregates in soils broken down due to the increase of tillage intensity, then forming a large number of small aggregates with relatively low organic carbon content and free organic matter particles. Free organic matter particles have poor stability and are easy to degradation, thereby causing the loss of SOC [33], [34]. In our study, at 0–7 cm soil depth, soil EOC under plowing tillage was lower than rotary tillage (Table 2). The reason could be attributed to the tillage method. Tillage increases the effect of drying–rewetting and freezing-thawing on soil, which increases macroaggregate susceptibility to disruption [21], [35], [36], and accelerates the labile organic C mineralization and SOM degradation, thus increasing the loss of EOC [14], [37]. At 7–14 cm, rotary tillage had higher soil EOC and DOC than plowing tillage, but lower at 14–21 cm soil depth, indicating that tillage affected the vertical distribution of EOC and DOC (Table 2). The difference in soil condition after plowing tillage or rotary tillage affects the rate of straw decomposition, thereby resulting in a difference in the soil nutrient accumulation [38]. Similarly, Liu et al. [39] have found that SOM content under plowing and rotary tillage at deeper soil both were higher than that of the upper soil. The reason might be that rotary tillage and plowing tillage mixed crop straw into the deeper soil layer, making SOM well-distributed at different depths [40].

Carbon input can be increased by adopting straw return in cropland [14]. Fresh residues are C source for microbial activity and nucleation centers for aggregation when returned to cropland. The enhanced microbial activity induces the binding of residue and soil particles into macroaggregates [34], [41], which could increase aggregates stability, fix the unstable C, thus improving the concentration of SOC [42] and increasing C sequestration [14]. In our research, straw return had significantly higher soil TOC and labile organic carbon fractions contents at the three soil depths than no crop straw return (Table 2). Soil TOC and labile organic C fractions in both rice and wheat straw return treatments were higher than only wheat or rice straw return (Table 2), indicating that straw return plays an very important role in increasing soil TOC and labile organic C fractions. Similar observations have been reported by other researchers [43]–[45]. At the three depths, soil TOC in the treatment of only wheat straw return in rice season was higher than only rice straw return in wheat season, moreover, the difference was significant (*p*<0.05) at 7–14 cm (Table 2). This was related to the relatively near-surface higher water content and favorable soil temperature during the rice growing season, resulting in relatively fast straw decomposition [46]. The decomposition of wheat straw provides enough energy and carbon source for soil microorganisms, thus increases the microorganisms’ activities. Alternatively, after wheat straw return, the high temperature and humid conditions accelerates the reduction of the C/N ratio of the straw, allowing for sufficient decomposition. More nutrients are released and utilized by the crops, which therefore lightens the pressure of burning straw and improves the soil quality [47]. According to table 1, the study showed that MBC was affected by the straw return factor with an affecting force of 95.95% at 0–7 cm depth and 97.83% at 7–14 cm depth. The probable explanation maybe that crop residue might enter the labile C pool, provide substrate for the soil microorganisms, and contribute to the accumulation of labile C [48].

In our study, PD had the highest content of soil TOC at all the three soil depths (Fig. 1). The reason might be that plowing tillage made the soil and straw in the plow layer turned over quarterly, which increased the stability of the TOC content at each soil layer [49]. In addition, the rice and wheat straw were both returned under PD treatment from 2009, plowing tillage made much SOM enter into the soil and accumulate [45]. However, Tian et al. [50] found that rotary tillage with straw return had higher SOC than plowing tillage with straw return at 0–10 cm soil depth in wheat field. The diverse results might be due to the different regional climate, soil type, crop rotation and the length of study [22]. In this study, at upper soil layer, the interaction effect between tillage and straw return was not significant, but generally increased with an increase in soil depth (Table 3). Rajan et al [2] also found that single effect of residue application was not significant but its significance became apparent after its interaction with tillage system.

In our study, soil labile organic C fractions were significantly and positively correlated with TOC concentrations at 0–21 cm soil depth (Table 1). Such correlations suggested that TOC was a major determinant of soil labile organic C fractions. MBC, DOC and EOC were also significantly and positively correlated with each other in this study (Table 1). The results were consistent with Chen et al. [14], who reported similar correlations between soil TOC, labile organic C fractions (MBC, DOC, particulate organic C, EOC and hot-water extractable C), and macroaggregate C within 0–15 cm depth. Dou et al. [51] also observed the same results. MBC is the living part of SOM, which plays an important role in maintenance of soil fertility [52]. It serves as a sensitive indicator of change and future trends in organic matter level [53]. Dissolved organic C consists of organic compounds present in soil solution, acts as a substrate for microbial activity, and is the primary energy source for soil microorganisms [1]. Easily oxidizable C partly reflects enzymatic decomposition of labile SOC [54]. Therefore, it is not surprising to find the positive correlations among the labile C pools as they have a close association with each other.

## Conclusions

In this study, after 2 years of a rice-wheat rotation, soil TOC and labile organic C fractions in PR, PW, PD, and RR, RW, RD were all higher than PN and RN. PD and RD had more significant effects on EOC, DOC and MBC compared to other treatments at 0–21 cm depth. Soil TOC and labile organic C fractions were highly correlated with each other. Under short-term conditions, rice and wheat straw both return in rice-wheat rotation system can increase SOC content and improve soil quality in the Yangtze River Delta, which is a suitable agricultural practice in this region under rice-wheat cropping system.

## References

[1] Haynes RJ (2005) Labile organic matter fractions as central components of the quality of agricultural soils: an overview. Adv. Agron 85: 221–268.

[2] Rajan G, Keshav RA, Zueng-Sang C, Shree CS, Khem RD (2012) Soil organic carbon sequestration as affected by tillage, crop residue, and nitrogen application in rice–wheat rotation system. Paddy Water Environ 10: 95–102.

[3] Blair GJ, Lefory RDB, Lise L (1995) Soil carbon fractions based on their degree of oxidation and the development of a carbon management index for agricultural system. Aust. J. Agric. Res 46: 1459–1466.

[4] Ghani A, Dexter M, Perrott WK (2003) Hot-water extractable carbon in soils: a sensitive measurement for determining impacts of fertilization, grazing and cultivation. Soil Biol. Biochem 35: 1231–1243.

[5] Rudrappa L, Purakayastha TJ, Singh D, Bhadraray S (2006) Long-term manuring and fertilization effects on soil organic carbon pools in a Typic Haplustept of semi-arid sub-tropical India. Soil Till. Res 88: 180–192.

[6] Yang CM, Yang LZ, Zhu OY (2005) Organic carbon and its fractions in paddy soil as affected by different nutrient and water regimes. Geoderma 124: 133–142.

[7] Baker JM, Ochsner TE, Venterea RT, Griffis TJ (2007) Tillage and soil carbon sequestration–what do we really know? Agric Ecosyst Environ 118, 1–5.

[8] Hernanz JL, L’opez R, Navarrete L, S’anchez-Gir’on V (2002) Long-term effects of tillage systems and rotations on soil structural stability and organic carbon stratification in semiarid central Spain. Soil Till. Res 66 (2): 129–141.

[9] Shan YH, Yang LZ, Yan TM, Wang JG (2005) Downward movement of phosphorus in paddy soil installed in large-scale monolith lysimeters. Agr. Ecosyst. Environ 111 (1–4): 270–278.

[10] Zhang ZJ (1998) Effects of long-term wheat-straw returning on yield of crop and soil fertility. Chinese Journal of Soil Science 29(4): 154–155.

[11] Sun X, Liu Q, Wang DJ, Zhang B (2007) Effect of long-term application of straw on soil fertility. Chinese Journal of Eco-Agriculture 16(3): 587–592.

[12] West TO, Post WM (2002) Soil organic carbon sequestration rates by tillage and crop rotation. Soil Science Society of American Journal 66: 1930–1946.

[13] Liu SP, Nie XT, Zhang HC, Dai QG, Huo ZY, et al (2006) Effects of tillage and straw returning on soil fertility and grain yield in a wheat-rice double cropping system. Transactions of the CSAE 22(7): 48–51.

[14] Chen HQ, Hou RX, Gong YS, Li HW, Fan MS, et al (2009) Effects of 11 years of conservation tillage on soil organic matter fractions in wheat monoculture in Loess Plateau of China. Soil Till. Res 106: 85–94.

[15] Wang DD, Zhou L, Huang SQ, Li CF, Cao CG (2013) Short-term Effects of Tillage Practices and Wheat-straw Returned to the Field on Topsoil Labile Organic Carbon Fractions and Yields in Central China. Journal of Agro-Environment Science 32(4): 735–740.

[16] Miller AJ, Amundson R, Burke IC, Yonker C (2004) The effect of climate and cultivation on soil organic C and N. Biogeoche mistry. 67: 57–72.

[17] Diekow J, Mielniczuk J, Knicker H, Bayer C, Dick DP, et al.. (2005) Soil C and N stocks as affected by cropping systems and nitrogen fertilisation in a southern Brazil Acrisol managed under no-tillage for 17 years. Soil and Tillage Research 81, 87–95.

[18] Galantini JA, Senesi N, Brunetti G, Rosell R (2004) In fluence of texture on organic matter distribution and quality and nitrogen and sulphur status in semiarid Pampean grassland soils of Argentina. Geoderma 123, 143–152.

[19] Ouédraogo E, Mando A, Stroosnijder L (2006) Effects of tillage, organic resources and nitrogen fertiliser on soil carbon dynamics and crop nitrogen uptake in semi-arid West Africa. Soil and Tillage Res 91: 57–67.

[20] Yamashita T, Feiner H, Bettina J, Helfrich M, Ludwig B (2006) Organic matter in density fractions of water-stable aggregates in silty soils: effect of land use. Soil Biology and Biochemistry 38: 3222–3234.

[21] Paustian K, Collins HP, Paul EA (1997) Management controls in soil carbon. In: Paul, E.A., Paustian, K.A., Elliott, E.T., Cole, C.V. (Eds). Soil Organic Matter in Temperate Ecosystems: Long Term Experiments in North America. RC, Boca Raton, FL 15–49.

[22] Puget P, Lal R (2005) Soil organic carbon and nitrogen in a Mollisol in central Ohio as affected by tillage and land use. Soil Till. Res 80, 201–213.

[23] Ding LL, Cheng H, Liu ZF, Ren WW (2013) Experimental warming on the rice-wheat rotation agro-ecosystem. Plant Science Journal 31(1): 49–56.

[24] Editorial Board of China Agriculture Yearbook (2012) China Agriculture Yearbook 2009, Electronic Edition. China Agriculture Press, Beijing, China.

[25] Hao JH, Ding YF, Wang QS, Liu ZH, Li GH, et al (2010) Effect of wheat crop straw application on the quality of rice population and soil properties. Journal of Nanjing Agricultural University 33(3): 13–18.

[26] Zhu LQ, Zhang DW, Bian XM (2011) Effects of continuous returning straws to field and shifting different tillage methods on changes of physical-chemical properties of soil and yield components of rice. Chinese Journal of Soil Science 42(1): 81–85.

[27] Liu SP, Nie XT, Zhang HC, Dai QG, Huo ZY, et al (2006) Effects of tillage and straw returning on soil fertility and grain yield in a wheat-rice double cropping system. Transactions of the CSAE 22(7): 48–51.

[28] Lu RK (1999) Soil agricultural chemistry analysis. China’s agricultural science and technology press, 106–110.

[29] Jiang PK, Xu QF, Xu ZH, Cao ZH (2006) Seasonal changes in soil labile organic carbon pools within a Phyllostachys praecox stand under high rate fertilization and winter mulch in subtropical China. For. Ecol. Manage 236: 30–36.

[30] Vance F, Brookes P, Jenkinson D (1987) Microbial biomass measurements in forest soils: the use of the chloroform fumigation-incubation method in strongly acid soils. Soil Biochem 19: 697–702.

[31] Leng SC (1992) Biological Statistic and Field Experimental Design. Beijing: China Radio& Television Press (in chinese).

[32] Duan HP, Niu YZ, Bian XM (2012) Effects of tillage mode and straw return on soil organic carbon and rice yield in direct seeding rice field. Bulletin of Soil and Water Conservation 32(3): 23–27.

[33] Yang JC, Han XG, Huang JH, Pan QM (2003) The dynamics of soil organic matter in cropland responding to agricultural practices. Acta Ecologica Sinica 23(4): 787–796.

[34] Six J, Elliott ET, Paustian K (1999) Aggregate and soil organic matter dynamics under conventional and no-tillage systems. Soil Sci. Soc. Am. J. 63, 1350–1358.

[35] Beare MH, Hendrix PF, Coleman DC (1994) Water-stable aggregates and organic matter fractions in conventional-tillage and no-tillage soils. Soil Sci. Soc. Am. J. 58, 777–786.

[36] Mikha MM, Rice CW (2004) Tillage and manure effects on soil and aggregate-associated carbon and nitrogen. Soil Sci. Soc, AM. J 68: 809–816.

[37] Wang J, Zhang RZ, Li AZ (2008) Effect on soil active carbon and C cool management index of different tillages. Agricultural Research in the Arid Areas 26(6): 8–12.

[38] Li XJ, Zhang ZG (1999) Influence on soil floods properties of mulching straws and soil-returning straw. Territory and Natural Resources Study 1: 43–45.

[39] Liu DY (2009) Physiological and ecological mechanism of stable and high yield of broadcasted rice in paddy field with high standing-stubbles under no-tillage condition. PhD: Sichuan Agricultural University.

[40] Gao YJ, Zhu PL, Huang DM, Wang ZM, Li SX (2000) Long-term impact of different soil management on organic matter and total nitrogen in rice-based cropping system. Soil and Environmental Sciences 9(1): 27–30.

[41] Jastrow JD (1996) Soil aggregate formation and the accrual of particulate and mineral associated organic matter. Soil Biol. Biochem. 28, 656–676.

[42] Govaerts B, Sayre KD, Lichter K, Dendooven L, Deckers J (2007) Influence of permanent raised bed planting and residue management on physical and chemical soil quality in rain fed maize/wheat systems. Plant Soil 291, 39–54.

[43] Stockfisch N, Forstreuter T, Ehlers W (1999) Ploughing effects on soil organic matter after twenty years of conservation tillage in Lower Saxony, Germany. Soil Till. Res 52: 91–101.

[44] Chen SH, Zhu ZL, Liu DH, Shu L, Wang CQ (2008) Influence of straw mulching with no-till on soil nutrients and carbon pool management index. Plant Nutrition and Fertilizer Science 14(4): 806–809.

[45] Song MW, Li AZ, Cai LQ, Zhang RS (2008) Effects of different tillage methods on soil organic carbon pool. Journal of Agro-Environment Science 27(2): 622–626.

[46] Zuo YP, Jia ZK (2004) Effect of soil moisture content o n straw decomposing and its dynamic changes. Joural of Northwest Sci-Tech University of Agri. and For. (Nat. Sci. Ed.) 32(5): 61–63.

[47] Dai ZG (2009) Study on nutrient release characteristics of crop residue and effect of crop residue returning on crop yield and soil fertility. PhD: Huazhong Agricultural University (in Chinese).

[48] Li CF, Yue LX, Kou ZK, Zhang ZS, Wang JP, et al (2012) Short-term effects of conservation management practices on soil labile organic carbon fractions under a rape–rice rotation in central China. Soil Till, Res 119: 31–37.

[49] Zhang P, Li H, Jia ZK, Wang W, Lu WT, et al (2011) Effects of straw returning on soil organic carbon and carbon mineralization in Semi-arid areas of southern Ningxia, China. Journal of Agro-Environment Science 30(12): 2518–2525.

[50] Tian SZ, Ning YT, Wang Y, Li HJ, Zhong WL, et al (2010) Effects of different tillage methods and straw-returning on soil organic carbon content in a winter wheat field. Chinese Journal of Applied Ecology 21(2): 373–378. 20462008

[51] Dou FG, Wright AL, Hons FM (2008) Sensitivity of labile soil organic carbon to tillage in wheat-based cropping systems. Soil Sci. Soc. Am. J. 72: 1445–1453.

[52] Wu TY, Schoenau JJ, Li FM, Qian PY, Malhi SS, et al (2004) Influence of cultivation and fertilization on total organic carbon and carbon fractions in soils from the Loess Plateau of China. Soil & Till, Res 77: 59–68.

[53] Gregorich EG, Ellert BH, Gregorich EG, Carter MR, Monreal CM, et al (1994) Towards a minimum data set to assess soil organic matter quality in agricultural soils. Can. J. Soil Sci 74: 367–385.

[54] Loginow W, Wisniewski W, Gonet SS, Ciescinska B (1987) Fractionation of organic carbon based on susceptibility to oxidation. Pol. J. Soil Sci 20: 47–52.

